# Multilevel Mapping of Sexual Dimorphism in Intrinsic Functional Brain Networks

**DOI:** 10.3389/fnins.2019.00332

**Published:** 2019-04-05

**Authors:** Nina de Lacy, Elizabeth McCauley, J. Nathan Kutz, Vince D. Calhoun

**Affiliations:** ^1^Department of Psychiatry and Behavioral Sciences, University of Washington, Seattle, WA, United States; ^2^Department of Applied Mathematics, University of Washington, Seattle, WA, United States; ^3^Mind Research Network, Albuquerque, NM, United States; ^4^Department of Electrical and Computer Engineering, University of New Mexico, Albuquerque, NM, United States

**Keywords:** intrinsic networks, ICA, sex-related differences, functional MRI, male, female

## Abstract

Differences in cognitive performance between males and females are well-described, most commonly in certain spatial and language tasks. Sex-related differences in cognition are relevant to the study of the neurotypical brain and to neuropsychiatric disorders, which exhibit prominent disparities in the incidence, prevalence and severity of symptoms between men and women. While structural dimorphism in the human brain is well-described, controversy exists regarding the existence and degree of sex-related differences in brain function. We analyzed resting-state functional MRI from 650 neurotypical young adults matched for age and sex to determine the degree of sexual dimorphism present in intrinsic functional networks. Multilevel modeling was pursued to create 8-, 24-, and 51-network models of whole-brain data to quantify sex-related effects in network activity with increasing resolution. We determined that sexual dimorphism is present in the majority of intrinsic brain networks and affects ∼0.5–2% of brain locations surveyed in the three whole-brain network models. It is particularly common in task-positive control networks and is pervasive among default mode networks. The size of sex-related effects varied by network but can be moderate or even large in size. Female > male effects were on average larger, but male > female effects spread across greater network territory. Using a novel methodology, we mapped dimorphic locations to meta-analytic association test maps derived from task fMRI, demonstrating that the neurocognitive footprint of intrinsic neural correlates is much larger in males. All results were replicated in a motion-matched sub-sample. Our findings argue that sex is an important biological variable in human brain function and suggest that observed differences in neurocognitive performance have identifiable intrinsic neural correlates.

## Introduction

Research in human neuroscience and psychology has described many differences in the performance of neurocognitive tasks between males and females. More recently, a newer generation of studies has re-examined these findings in a more nuanced fashion, attempting to take into account the influence of potential co-varying factors such as gender identification and socialization. While this latter work has de-emphasized the wide scope of earlier findings, some types of cognitive tasks have been confirmed to show significant differences in performance between males and females. Broadly, males tend to exhibit superior performance in selected spatial and visuospatial tasks and are somewhat disproportionately represented among high performers in mathematics, whereas females perform better at reading and certain tasks of verbal fluency, recognition memory, and episodic memory (Miller and Halpern, 2014). However, differences may be quite task specific. For example, men reliably show superior performance on tasks of mental rotation but only inconsistently in mental folding, and the performance gap in mental rotation is wider for 3-dimensional versus (vs.) 2-dimensional objects (Voyer et al., 1995). In some types of tasks differences in performance are striking and appear to straddle cultural and educational environments: in a sample of 1.5 million children across 75 nations, girls consistently outperformed boys on reading (Stoet and Geary, 2013). Considerable variation also exists at the level of the individual.

Sex-related differences in cognition are of interest not only for the study of the neurotypical brain, but also in the context of neuropsychiatric disorders. Sex profoundly influences the prevalence, incidence and severity of various neuropsychiatric disorders. Well-known examples include the increased prevalence of attention deficit hyperactivity disorder (ADHD) and autism in males, vs. higher lifetime rates of depression and anxiety in females. The incidence of anxiety disorders accelerates faster in adolescent females, whereas schizophrenia onsets earlier in males, with a more severe course. Sex-related differences are also present in the diseases of senescence, with Alzheimer’s disproportionately affecting females, but Parkinson’s disease and Lewy body dementia being 2-4x more common in men (Podcasy and Epperson, 2016). While altered brain function associated with diagnosis has been described in most neuropsychiatric disorders, sex-related differences appear to further mediate disrupted function, suggesting interactions between sex and the requisite developmental or disease process. For example, diagnosis × sex interactions in brain function have been identified in autism (Yang and Lee, 2018) and our own work in ADHD (de Lacy et al., 2018). Similar phenomena occur in aging and dementia. For example, rates of cognitive decline with aging differ between males and females, and sex-related differences have been reported in the cognitive impairment attributable to Alzheimer’s disease (Li and Singh, 2014).

The continuous development of non-invasive imaging technologies with good spatial resolution such as magnetic resonance imaging (MRI) has enabled the investigation of sex-related differences in human brain structure and function, building bridges between observed differences in cognitive performance and their potential neural correlates. Perhaps the most robust findings have been of a greater proportion of gray matter in females as compared to white matter and larger absolute brain volume in males, even after correcting for body size (Gur et al., 1999). The former finding is present even in childhood. Other work suggests that dimorphism also exists in cortical thickness and gyrification (Im et al., 2006; Mutlu et al., 2013). A recent large study of older adults from the UK brain biobank confirmed overall thicker cortices in women, but more variation among men in regional volumes, gyrification and cortical thickness (Ritchie et al., 2018). Further, a number of studies indicate that volumetric and white-matter differences between men and women are regionally specific (Sacher et al., 2013; Guadalupe et al., 2017) and include differences in the topology of anatomic (white matter) connectivity (Gong et al., 2011).

Given the close relationship between brain function and cognitive performance, identifying the functional neural correlates of sex-related cognitive differences is of considerable interest. The latter have been explored using functional MRI (fMRI). In fMRI during task performance, sex-related differences have been observed paralleling the psychological literature, such as visuospatial tasks (Gur et al., 2000). In the task-free state, intrinsic or spontaneous brain activity is recorded *in vivo* using resting-state fMRI (rsfMRI). This activity is spatio-temporally organized, and replicable macroscale intrinsic neural networks have been identified with specific neurocognitive associations (Laird et al., 2011). Considerable controversy exists regarding dimorphism in intrinsic networks. For example, several studies have described sex-related differences in fronto-parietal, cingulo-opercular and temporal connections in typically developing adults (Biswal et al., 2010; Zuo et al., 2010) or individual networks such as the salience and default mode network (DMN) in healthy aging (Jamadar et al., 2018). Conversely, others have found no differences between men and women in certain prominent intrinsic networks and suggested sex need not be modeled as a variable in rsfMRI studies (Weissman-Fogel et al., 2010). Given the large and growing number of rsfMRI studies, this is a pressing research question.

The relative ease of acquisition of rsfMRI render it a tractable medium for examining brain function across ages, species and cognitive levels. Further, macroscale intrinsic networks observed in the resting condition are similar to those detected during task performance (Smith et al., 2009), being theorized to represent historical activity patterns and/or task activation ‘templates.’ It may therefore be hypothesized that intrinsic brain networks may have differences in their spatial characteristics in men and women that map onto those cognitive abilities exhibiting sex-related differential performance in the population. One way to examine these relationships is to analyze differences in intrinsic networks and correlate them to ex-scanner or in-scanner task performance. Experimentally, these approaches are challenged by the difficulties inherent in constructing the requisite required large array of cognitive tasks and administering them to a sufficiently well-powered group of subjects. To address this, we recently developed a novel methodology allowing *in silico* mapping of statistical effects identified in intrinsic networks onto meta-analytic association test maps of neurocognitive functions. This approach allows the construction of a computational ‘bridge’ from brain networks observed during rsfMRI to neurocognitive maps derived from hundreds of task fMRI experiments.

To advance the debate on sex-related differences in intrinsic brain networks, we asked whether we could isolate male > female (M > F) and female > male (F > M) effects in intrinsic brain networks in a large sample of typically developing young adults matched for age and sex, and how these effects related to brain maps associated with nine neurocognitive functions with good evidence of sex-related performance differences. As noted above, sex-related performance differences have also historically been detected in many other cognitive tasks, albeit less consistently or less frequently reported. For comparison purposes, we therefore also elected to survey a selection of cognitive control functions, where there is a less robust evidence base for sex-related performance differences. Given the existing controversy in this field, we formulated a design where intrinsic brain networks were obtained using independent component analysis (ICA), a popular and very well-established (Calhoun and Adali, 2012) data-driven method of discovering networks, and pursued multilevel modeling. Here, three separate whole-brain models of 8, 24, and 51 networks were formulated to describe functional brain organization with increasing refinement and to assess whether major findings were stable across different model orders, since there is currently no standard method to determine the optimal number of intrinsic networks when modeling whole-brain rsfMRI. Multivariate modeling of M > F and F > M effects was applied in 8-, 24-, and 51-network models, where the data-driven functional brain parcellation estimated with ICA was the same for all participants within each model. Further, we performed a replication analysis in a subsample of subjects matched for head motion.

## Materials and Methods

### Data

This study uses data from the Brain Genomics Superstruct project, collected from > 3,000 individuals in the Boston community enrolled in studies of normal brain function or as controls in clinical studies^1^. From this larger initiative, the originators formed and released a repository in 2015 comprising demographic, MRI and behavioral data from a subset of 1570 healthy young adults ages 18–35, where age was specified within 2-year bins. For example, the 19-year-old bin includes subjects aged 18 and 19 at the time of scanning. Our study uses data from subjects in this latter sample, where the “dispersion of estimated IQ scores [was] positively shifted relative to the general population” but personality traits “have distributions that would be expected of a clinically screen population-based sample (Holmes et al., 2015). Of note, IQ scores were derived from Shipley-Hartford Age-Corrected T scores. The present study was deemed not human subjects research by the University of Washington Institutional Review Board.

### MRI Pre-processing

In this step, unprocessed rsfMRI data was processed with a standard SPM12 pipeline to prepare it for modeling with ICA. MRI scans were collected using matched 3T TIM Trio systems at Harvard University and Massachusetts General Hospital using vendor-supplied 12-channel head coils, on 5 different scanners. 124 volumes (6.12 min) of functional MRI were acquired with 47 slices, interleaved sequence, voxel size 3.0 mm × 3.0 mm × 3.0 mm and TR = 3 s. Resting state scans were acquired with participants instructed to “remain still, stay awake and keep their eyes open while blinking normally” (Holmes et al., 2015). A fixation cross was not employed. Full details of parameters may be obtained from the Brain Genomics Superstruct website^2^. The originators of the data kept scan time relatively short at 6.12 min to reduce the risk of movement. Prior work has also demonstrated that 5–7 min of rsfMRI is sufficient to obtain stable estimates of intrinsic networks (Fox et al., 2005; Van Dijk et al., 2010). In addition, extensive quality control was performed by the originators of the data, including screening for “artifacts, acquisition problems, processing errors and excessive motion with each image viewed on a per-slice basis along each principal axis” and data from 54 participants were excluded from release on this basis (Holmes et al., 2015). Slice-based temporal signal-to-noise ratio (sSNR) was also computed (Holmes et al., 2015) and 88 participants with sSNR < 100 were excluded from release, thus ensuring that all subjects in the present study have sSNR > 100. As recommended by the originators of the data, the first 4 volumes of each scan were removed to account for scanner equilibration effects, with 120 timepoints remaining. Subsequently, volumes were slice-time corrected to the middle volume, realigned to the first volume, resliced, coregistered, and normalized to the functional template and smoothed at 6 mm full width half maximum using standard algorithms in SPM12. After processing, data were submitted to quality control to assess the quality of the normalization and degree of subject motion by computing (1) spatial regression between each normalized functional image and a group mask constructed from all subjects and (2) root mean square difference of volume N to volume N+1, also known as DVARS (Christodoulou et al., 2013; Power et al., 2014). All subjects had > 85% correspondence between their normalized image and the group mask with one exception. Normalization for this subject proved uncorrectable and this participant was eliminated from further consideration.

### Subject Sample Construction

In this step, two samples of participants were prepared from the total of 1569 GSP subjects remaining after pre-processing (see MRI Pre-processing). The first was a 670-subject sample matched for age and sex, the second a 535-subject sample additionally matched for motion. The 670-subject sample for this study was constructed by selecting right-handed subjects with estimated IQ scores available, and then sex-matching within age bins. Subject demographics may be viewed in Table 1 and a list of subjects inspected in Supplementary Table 1. In this sample, there was a significant difference in head motion as measured by DVARS between males and females (*p* = 6.995 × 10^-9^), with males having higher average scores. Accordingly, we also created a sub-sample of 534 subjects that were similarly all right-handed and matched for age and sex but were also matched for head motion as defined by DVARS score. A list of the 136 subjects removed to create this motion-matched sample is in Supplementary Table 2. There was no significant difference in estimated IQ between groups in the two samples. The terms ‘sex,’ ‘male’ and ‘female’ are used in this paper in accordance with the phenotypic nomenclature used by the Brain Genomics Superstruct project.

**Table 1:** Subject demographics.

	Male	Female
Number of subjects	335	335
Average estimated IQ	114.9	113.0
Age range	19–35	19–35

### Construction of Whole Brain Models of Intrinsic Networks

In this step, pre-processed rsfMRI data (see MRI Pre-processing) for the two subject groups (see Subject Sample Construction) was used to generate 8-, 24-, and 51 network models of brain function by submitting the pre-processed data to group spatial ICA (see Group Spatial ICA). Gray matter components were identified in each of the 3 models by taking the entire output (all components) of the group ICA and eliminating noise components (see Sorting Components From the Spatial ICA). The resulting gray matter components were thresholded in order to construct spatial maps of each intrinsic network representing the strongest and most consistent coactivations between brain regions within a network (see Construction of Intrinsic Functional Network Spatial Maps). These spatial maps were used to attribute the neurocognitive labels for each IN (see Functional Intrinsic Network Attribution and Grouping), and served as the inputs for the remainder of the analyses. This process was followed for each brain map in each of the 3 ICA models.

#### Group Spatial ICA

Using the pre-processed rsfMRI data (see MRI Pre-processing), we performed group spatial ICA using the Group ICA of fMRI Toolbox (GIFT) developed in our group, and widely used in ICA of fMRI (Calhoun et al., 2001; Calhoun and Adali, 2012). ICA is a popular method of providing data-driven functional brain parcellations in rsfMRI data and resultant sets of intrinsic networks for further analysis. Currently, no method exists to determine an optimal number of components/networks for any specific individual model. Rather, the number of components selected for a study is an analytic choice. Generally, higher model orders with more components provide more detailed views of brain function, i.e., more networks. The ultimate number of networks estimated by an ICA model depends therefore on (1) The initial number of components specified for the model minus (2) The number of noise components eliminated after model estimation. In the present study, we performed 3 ICA decompositions to test the sensitivity of results to model parameters and provide an increasingly detailed view of brain networks. Resting-state scans were first pre-whitened followed by a subject-specific data reduction principal components analysis retaining 20, 50, and 110 principle components (PCs) respectively, with the objective of stabilizing back reconstruction and retaining maximum variance at the individual level. Group ICA decompositions were then performed with 15, 40, and 100 components respectively using the Infomax algorithm run 10 times with random initialization using ICASSO (Himberg et al., 2004; Li et al., 2007). Aggregate spatial maps were estimated as the centrotypes of component clusters to reduce sensitivity to initial algorithm parameters. Single-subject images were concatenated in time to perform the single group ICA estimation and subject specific spatial maps estimated using back reconstruction (Erhardt et al., 2011) with the group information guided ICA (GIG-ICA) algorithm (Du et al., 2016), an approach which we have shown well-captures individual subject variability (Allen et al., 2012). GIG-ICA estimates single-subject images and timecourses from the single group ICA estimation, thereby allowing individual variation in spatial maps constructed from each component (see below). The resulting independent components were scaled by converting each subject component image and the time course to *z*-scores.

#### Sorting Components From the Spatial ICA

Using the output of all components from each of the 3 ICA decompositions (see Group Spatial ICA), we sorted components into gray-matter networks vs. artefactual noise components with a combination of expert visual inspection by NdL and VC, and quantitative metrics in order to isolate gray-matter or neural components. To do this we computed the quantitative spectral metrics of (1) Fractional amplitude of low frequency fluctuations and (2) Dynamic range (Allen et al., 2011) for every component. The former is the ratio of the integral of spectral power below 0.10 Hz to the integral of power between 0.15 and 0.25 Hz. Dynamic range is the difference between the peak power and minimum power at frequencies to the right of the peak. Generally, components representing gray matter have higher values in these metrics, while artefactual components (such as signals accruing from cerebrospinal fluid, vascular pulsations, white matter or head motion) have lower values, though there are currently no absolute cut-off points for inclusion or exclusion. Components were inspected by NdL and VC and those with poor overlap with cerebral gray matter or low spectral metrics were discarded. Where components were deemed to be ‘mixed’ components containing a probable mixture of gray-matter signal and noise, we discarded these components to promote a more conservative approach with higher-quality networks. In particular, sub-cortical/cerebellar components tend in our empirical experience to appear as ‘mixed’ components. For example, in the present analysis we accepted only a single cerebellar network in the 24- and 51-network models. We retained 8 components from the 15-component ICA, 24 from the 40-component and 51 from the 100-component ICA, each considered a set of functional intrinsic brain networks (INs). Thus, 40–50% of the components were discarded from each component set, a ratio in line with comparable studies using ICA in other data samples (Allen et al., 2011; Rashid et al., 2014; de Lacy et al., 2017).

#### Construction of Intrinsic Functional Network Spatial Maps

**FIGURE 1:**
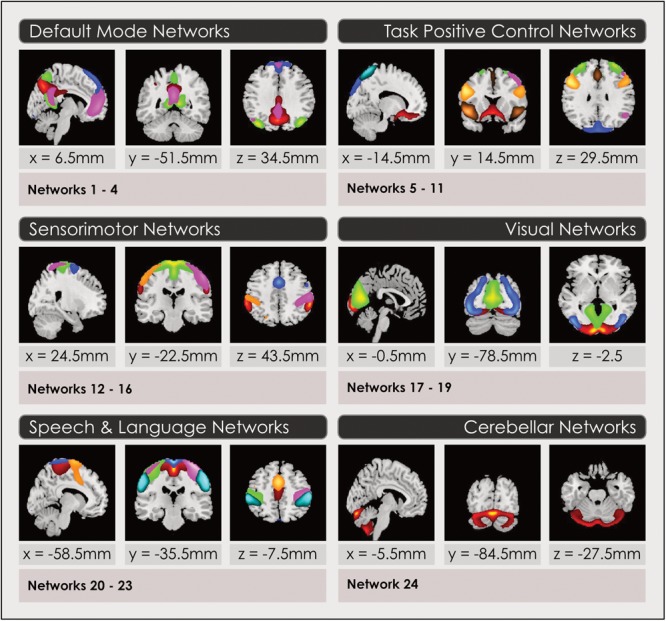
Intrinsic networks grouped by neurocognitive function for 24-network model. Group spatial maps are displayed in 2-dimensional format for a representative slice, for the 24-network model of intrinsic networks grouped by neurocognitive function. Spatial maps are created by thresholding gray-matter components from the group ICA at (mean + 4 standard deviations). Neurocognitive attributions were made using three methods described in the Section “Functional Intrinsic Network Attribution and Grouping.” Readers may explore 3-dimensional maps of each network in Neurovault at https://neurovault.org/collections/4030/ (8-network model), https://neurovault.org/collections/4031/ (24-network model) and https://neurovault.org/collections/4032/ (51-network model), where networks are labeled with numbers and their neurocognitive attributions.

After the sorting process (see Sorting Components From the Spatial ICA) we constructed a spatial map for each gray-matter IN that had been retained to select voxels that represented the strongest and most consistent coactivations within each IN, by performing a voxelwise one-sample *t*-test on the individual subject timecourses and thresholding individual voxels at (mean + 4 standard deviations), again following an established pipeline (Allen et al., 2011) using GIFT. Thus, these spatial maps represent the brain regions most associated with each component’s timecourse, instantiated in thresholded brain maps. This procedure enabled us to construct a group spatial map for each of the INs assembled from the relevant individual subject timecourses, in each of the model orders. These spatial maps were used to attribute the neurocognitive labels for each IN, and served as the inputs for the remainder of the analyses. Three-dimensional renderings of the resulting 3 sets of intrinsic networks may be inspected in Neurovault at https://neurovault.org/collections/4030/ (8-network model), https://neurovault.org/collections/4031/ (24-network model) and https://neurovault.org/collections/4032/ (51-network model). Each intrinsic network is labeled with its attributed neurocognitive function and numbers, that correspond to Figure 1, 3, 4, 6, 7.

#### Functional Intrinsic Network Attribution and Grouping

The primary neurocognitive function of each IN spatial map constructed in Section “Construction of Intrinsic Functional Network Spatial Maps” was attributed by visual inspection and quantitative comparisons using three methods in order to assign it an associated neurocognitive function and label. The output of this final step of the ICA process was 3 sets of thresholded spatial maps of intrinsic networks with associated neurocognitive labels. The subsequent statistical analysis to identify sex-related effects and map these to neurocognitive functions was performed on these 3 models of whole-brain function. To label each IN, we first determined the coordinates in Montreal Neurologic Space (MNI) associated with peak intensities for each IN in each of the 3 sets of maps. The top 3 co-ordinates were compared with the literature. We found multiple literature-based confirmatory sources that gave specific Talairach or MNI coordinates and associated these with network labels for all networks in the task-positive network group, the DMN and primary sensorimotor and visual networks (Fox et al., 2005; Dosenbach et al., 2006, 2007; Seeley et al., 2007; Smith et al., 2009; Laird et al., 2011; Spreng et al., 2013; Vernet et al., 2014) but not for INs in the subcortical or speech/language groups. Secondly, the top 5 spatial locations in each IN were examined using the Brodmann Interactive Atlas^3^. Thirdly, network correlations with association test maps of regional activations associated with specific neurocognitive functions were inspected in Neurosynth (Yarkoni et al., 2011). Attributions using the third method may be explored by readers by loading a spatial map in Neurovault and accessing the ‘Cognitive Decoding’ function.

### Statistical Analysis to Identify Sex-Related Effects in IN Spatial Maps

In this step, we used the group ICA output of 3 sets of labeled, thresholded spatial maps of INs (see Construction of Whole Brain Models of Intrinsic Networks) as the substrate for a multivariate statistical analysis aimed at finding sex-related differences in each IN in each of the 3 models of whole brain function. We first performed a multivariate analysis of covariance (MANCOVA) using the MANCOVAN toolbox and an established method (Allen et al., 2011) in GIFT, to compare the effects of sex with other possible predictors of variance in the 3 sets of network maps for (a) The original 670-subject sample and (b) The 534-subject motion-matched sample. To optimize for the large dimensions of the data but enable statistical testing at each voxel, predictors were submitted to the MANCOVA with an F-test at each iteration to produce a final reduced model for each outcome measure and network, before univariate testing of significant predictors was performed on the original model with correction for multiple comparisons (among all networks analyzed within a set) and false discovery rate (FDR) at α = 0.01. As detailed above, the GSP project acquired data on 5 different matched Siemens scanners and we controlled for scanner site in our analysis. We used sex, age bin, estimated IQ-level, scanner bin and DVARS measure as predictors for all three analyses. The effects of age, estimated IQ-level, DVARS and scanner bin were regressed from the analysis using the general linear model, to isolate the effects of sex. However, we retained all variables including scanner site to test for any residual effects on the statistical analyses. For example, we tested for residual DVARS × sex effects. Significant effects were computed for both positively correlated voxels in each network (F > M effect) and for negatively correlated voxels (M > F effect).

For each predictor that proved significant in the univariate analysis, the effect size (beta) was determined by computing connected voxel clusters (similarly to the bwlabeln function in MATLAB) and then calculating an average beta over the cluster of voxels. The fraction of the network map accounted for by each effect was determined by calculating the fraction of the total voxels in each network map represented by voxels with significant effects (significant voxels/total voxels). The size and fraction of both F > M and M > F effects were computed for each of the 3 IN sets in both subject groups.

### Mapping Significant Effects to Neurocognitive Functional Maps

In this final step, we used the output of the multivariate analysis (see Statistical Analysis to Identify Sex-Related Effects in IN Spatial Maps) which identified significant sex-related effects in each IN in each model as the input to a mapping process where we mapped effects of sex in each IN to neurocognitive functional maps using a method which we recently developed and published (de Lacy et al., 2018). The aim of this analysis was to identify and compare the cognitive ‘footprint’ of sex-related differences in IN function across 16 cognitive functions and see how this varied between sexes, cognitive functions and 8-, 24-, and 51-network models of brain function. First, effect maps were created to map voxels with significant (α < 0.01, corrected for FDR and multiple comparisons) F > M and M > F effects in the univariate analysis for each network. For example, a map of the effects of M > F in the right fronto-parietal network. Association test maps were created using custom code written in Python to access the Neurosynth (Yarkoni et al., 2011) database and analytic engine for each of the following terms: visuospatial; spatial; verbal; verbal fluency; semantic memory; rotation; recognition memory; reading and arithmetic. We selected these terms based on a qualitative review of the prior literature pertaining to significant performance differences between male and female subjects in psychological and neurocognitive performance (See, for example, the excellent review by Miller and Halpern (2014). In addition, we performed a comparison with a set of major cognitive control function terms: cognitive flexibility; goal selection; reaction time; response selection; selective attention; sustained attention and working memory. We accessed the entire database of task fMRI studies available in Neurosynth at the time of our analysis, which was performed between April and July 2018, prior to the recent Neurosynth update. Of note, Neurosynth recently changed the terminology used to refer to neurocognitive maps, now preferring the terms ‘uniformity test map’ and ‘association test map.’ At the time our analysis was performed the terms in use were ‘forward inference map’ and ‘reverse inference map.’ In this manuscript we use the updated term ‘association test map’ though at the time our analysis was performed these neurocognitive maps were referred to as ‘reverse inference maps.’ Limitations remain, reviewed below (See: Limitations).

Neurosynth association test maps are z-score fMRI activation maps derived from a database at the time of our analysis of > 11,000 studies in the neuroscience literature in task-based fMRI. Neurosynth^4^ uses text mining to identify terms of interest (e.g., “spatial”) within neuroscience articles occurring at a frequency of > 1/10,000 words, and extracts fMRI activation coordinates from tables in the corresponding article text. These term to activation mappings are used to construct the database. Automated meta-analysis is performed for a psychological term of interest (e.g., “recognition memory”) to construct a whole-brain association test map of the posterior probability of a term of interest occurring given activation at each voxel. This contrasts with forward inference maps such that are commonly obtained in task-based fMRI, or conventional meta-analyses, which often display the probability of brain activation given a task, or term. Therefore, association test maps may be conceptualized as meta-analytic maps identifying brain location activations, that are relatively more selective for the neurocognitive function of interest than forward inference maps. This procedure controls for the fact that many brain locations are implicated in multiple functions and are non-specifically activated in experiments. The process by which maps are generated by Neurosynth is wholly automated, and multiple validation techniques were applied by the original authors to compare results with manual techniques (Yarkoni et al., 2011). Overall, their results demonstrated that for broad domains of cognition, such as are considered in the present study, the composite Neurosynth algorithm extracts the majority of coordinates accurately to form the underlying database and produces results comparable in sensitivity and specificity to manual meta-analytic approaches. In the present study, we used custom Python code to access the Neurosynth database and generate association test maps corresponding to terms of interest, but otherwise all computational procedures were similar.

Custom code was written in MATLAB to identify locations (voxels) in the brain where individual effect maps were spatially coincident with activations in association test maps for each neurocognitive function. This code is available in GitHub at ninadelacy/effect-mapping. Every combination of significant F > M and M > F effects and association test maps was computed, to determine voxels that were present in both maps for each combination. To create aggregated maps of effects in each subject across each neurocognitive function, overlapping voxels from F > M and M > F were collected, and redundancies eliminated to determine only unique voxels. We calculated the relative numbers of brain locations implicated in each neurocognitive functional map for F > M and M > F by summing the unique voxels for each neurocognitive functional map and dividing into the relative proportions for each subject group.

## Results

### Sexual Dimorphism Was Present in the Majority of Intrinsic Functional Networks

**FIGURE 2:**
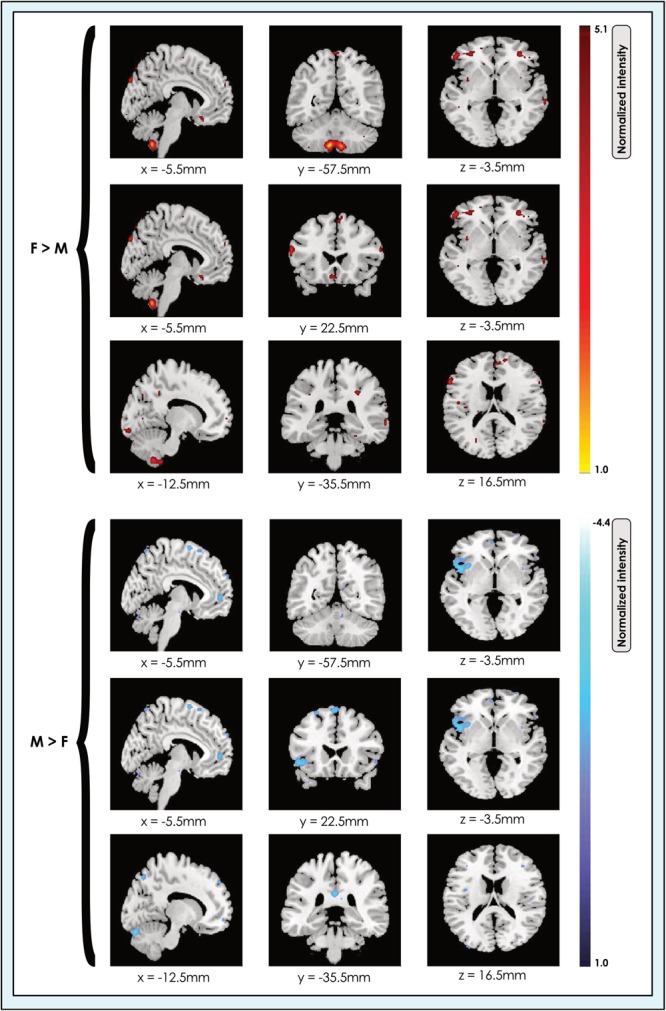
Locations of sex-related effects in a 24-network model of whole brain function in 670 neurotypical young adults. The effect of sex is shown for M > F and F > M in young adult functional brain networks (α < 0.01, corrected for multiple comparisons and false discovery rate). We present maps showing all dimorphic locations concatenated across networks in a 24-network whole-brain model in resting-state fMRI data from 670 neurotypical adults ages 19–35 matched for age and sex. We tested for significant sex-related differences at every voxel. Accordingly, both M > F (blue) and F > M effects (red-yellow) are possible within each network. Effects of each type are shown at three different slice locations with pairs of locations shown for each effect type to facilitate comparison. Readers may explore 3-dimensional effect maps available in Neurovault at https://neurovault.org/collections/4034/, where F > M effects are shown in red, and M > F effects in blue.

We determined the spatial location of significant sex-related effects in each of the 8-, 24-, and 51-network models (Figure 2).

**FIGURE 3:**
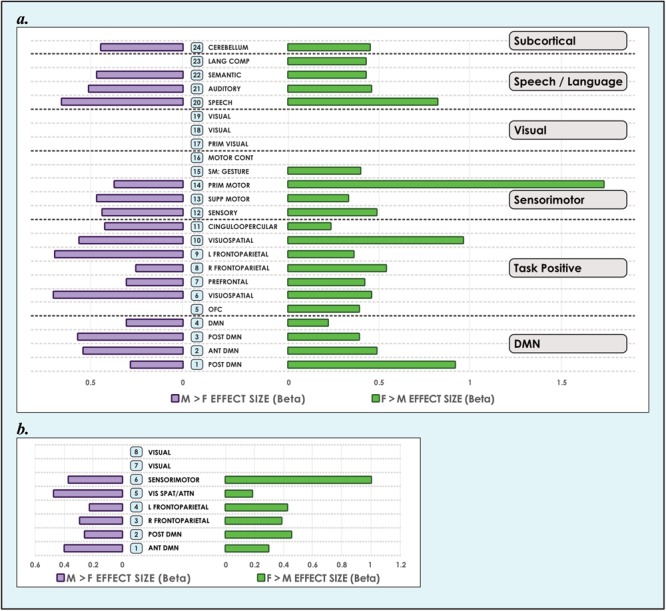
Sex-related effect sizes among intrinsic networks in 8- and 24-network models of brain function in 670 neurotypical young adults. The effect of sex is shown for M > F and F > M in young adult functional brain networks (α < 0.01, corrected for multiple comparisons and false discovery rate). We present both **(a)** 24-network and **(b)** 8-network whole-brain models estimated using 15- and 40-component independent component analyses respectively, performed on resting-state fMRI data from 670 neurotypical adults ages 18–35 matched for age and sex. We tested for significant sex-related differences at every voxel in each network. Accordingly, both M > F and F > M effects are possible within each network. Vis spat/attn, Visuospatial/Attention; L, Left; R, Right; Post, Posterior; Ant, Anterior; Lang Comp, Language Comprehension; Prim, Primary; Motor Cont, Motor Control; SM, Sensorimotor; Supp, Supplementary; OFC, Orbitofrontal; DMN, Default Mode Network. Network labels correspond with numbers and attributions for 3-dimensional maps available in Neurovault.

**FIGURE 4:**
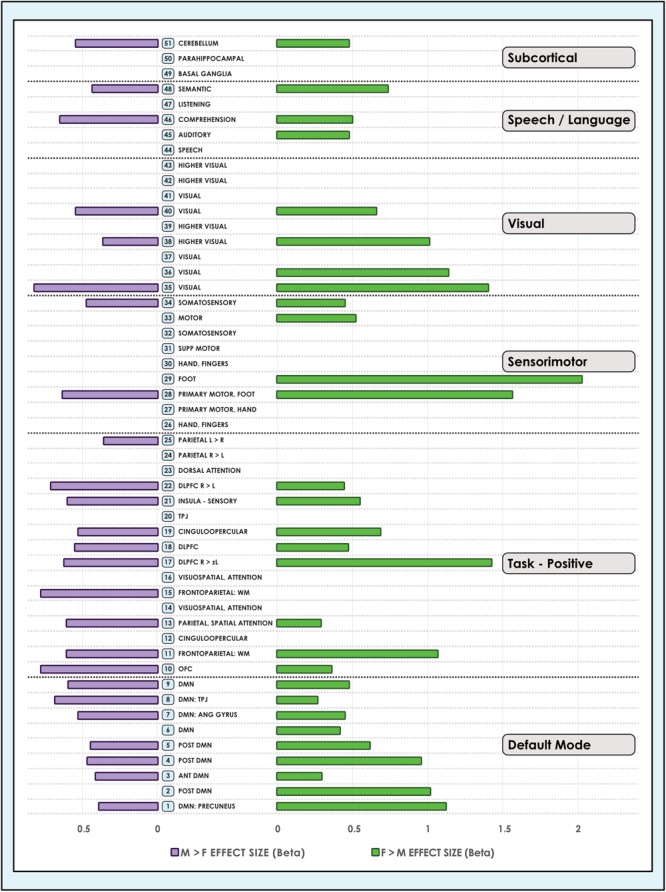
Sex-related effect sizes among intrinsic networks in a 51-network model of brain function in neurotypical young adults. The effect of sex is shown for M > F and F > M in young adult functional brain networks (α < 0.01, corrected for multiple comparisons and false discovery rate). Effect sizes are shown for a 51-network whole-brain model estimated using a 100-component independent component analysis performed on resting-state fMRI data from in 670 neurotypical adults ages 18–35 matched for age and sex. We tested for significant sex-related differences at every voxel in each network. Accordingly, both M > F and F > M effects are possible within each network. L, Left; R, Right; Post, Posterior; Ant, Anterior; SM, Sensorimotor; Supp, Supplementary; DLPFC, Dorsolateral Prefrontal Cortex; TPJ, Temporoparietal Junction; WM, Working Memory; OFC, Orbitofrontal; DMN, Default Mode Network; Ang Gyrus, Angular Gyrus. Network labels correspond with numbers and attributions for 3-dimensional maps available in Neurovault.

Our analyses revealed that significant (α < 0.01, corrected for multiple comparisons and false discovery rate) sex-related differences were widely present in intrinsic networks in each of the three models (Figure 3, 4), ranging from ∼60% in the 51-network model to >80% in the 24-network model (Supplementary Tables 3–5). In nearly all INs with sex-related differences, both types of effects (M > F and F > M) were present within the same network. Exceptions - where an effect of only one type was present in an individual network (i.e., F > M or M > F only) - were relatively rare. In these asymmetric cases, the unpaired effect typically occupied a relatively small proportion of the network, albeit these instances were more frequent as model order increased. Specifically, INs associated with language comprehension, gesture and the orbitofrontal cortex displayed only F > M effects in the 24-network model. In the 51-network model, the auditory, two motor, and two DMNs, and one of nine visual networks only displayed F > M effects, and a parietal network and frontoparietal network associated with working memory only M > F effects. In the motion-matched sample the number of effects increased overall, with nearly 90% of INs exhibiting significant (α < 0.01, corrected for multiple comparisons and false discovery rate) sex-related differences in the 8- and 24-network models, and >70% in the 51-network model (Supplementary Table 6).

Of note, occasional differences were observed among networks with similar neurocognitive functions. For example, among the speech and language group, we identified only F > M effects in an IN associated with language comprehension in the 24-network model, but both M > F and F > M effects in a similar IN in the 51-network model. In the motion-matched sample these asymmetries were typically reduced given the increased number of sex-related effects present.

### Sex Differences Were Pervasive in Default Mode Networks

Analyzing 3 model orders permitted the examination of sex-related effects in brain networks in increasing detail. For example, while one sensorimotor network was present in the 8-network model, 9 sensorimotor networks were present in the 51-network model, associated with differing functional emphases. A consistent finding was that both M > F and F > M effects were present in all default mode networks, regardless of model order (Figure 3, 4). The only exceptions were 2 default mode networks of the 9 present in the 51-network model, where only F > M effects were detected (Figure 4). However, in the motion-matched sample these exceptions were not present (Supplementary Table 5), and all default mode networks showed sex-related differences. This striking finding contrasts with other network types, where sex-related effects were less pervasive, particularly in the 51-network model. In particular, visual and sub-cortical networks exhibited a relative paucity of sex-related effects, excepting the cerebellum, and no dimorphism was detected in attentional networks. This latter finding is most clearly seen in the 51-network model, where subnetworks associated with attentional function were isolated such as the dorsal attention network, visual attention networks and the temporo-parietal junction (associated with the ventral attention network).

### Sex-Related Effects Were on Average Larger in Females, but Occupied More Brain Territory in Males

**FIGURE 5:**
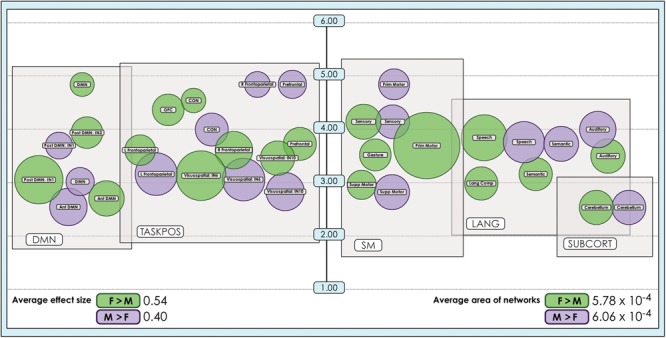
Comparison of size of sex-related effects in intrinsic networks and proportion of network affected by dimorphism in a 24-network model of brain function. The size of significant (α < 0.01, corrected for multiple comparisons and false discovery rate) effects of sex is shown for M > F and F > M in a 24-network model of functional brain networks estimated using independent component analysis in 670 neurotypical young adults matched for age and sex. Effect sizes (size of bubble) are compared to the fraction of the network map (position on vertical scale) affected by significant sex-related effects, computed as a percentage of all voxels analyzed from resting-state functional MRI. Vertical scale is a log_10_ scale. Average area is calculated as the arithmetic mean of fractions pertaining to each individual network. Vis spat/attn, Visuospatial/Attention; L, Left; R, Right; Post, Posterior; Ant, Anterior; Lang Comp, Language Comprehension; Prim, Primary; Motor Cont, Motor Control; SM, Sensorimotor; Supp, Supplementary; OFC, Orbitofrontal; DMN, Default Mode Network; CON, Cinguloopercular network. Network labels correspond with numbers and attributions for 3-dimensional maps available in Neurovault. Of note, the position of the bubbles relative to the *x*-axis has no quantitative meaning and has been set to support visual clarity.

**FIGURE 6:**
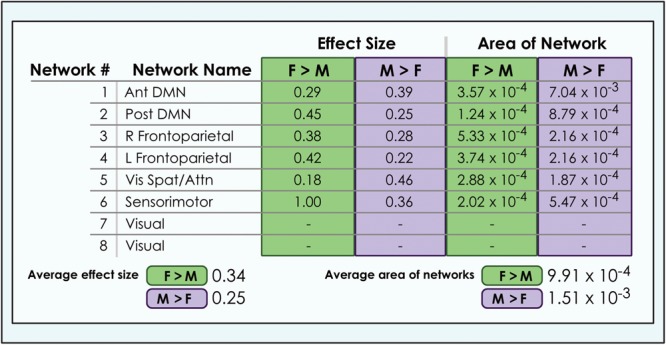
Comparison of size of sex-related effects in intrinsic networks and proportion of network affected by dimorphism in a 8-network model of brain function in 670 neurotypical young adults. The size of significant (α < 0.01, corrected for multiple comparisons and false discovery rate) effects of sex and proportion of network affected is shown for M > F and F > M in an 8-network model of functional brain networks. The fraction of each network map with sex-related effects was computed as a percentage of all voxels analyzed from resting-state functional MRI. Average area is calculated as the arithmetic mean of fractions pertaining to each individual network. Vis spat/attn, Visuospatial/Attention; L, Left; R, Right; Post, Posterior; Ant, Anterior; DMN, Default Mode Network. Network labels correspond with numbers and attributions for 3-dimensional maps available in Neurovault.

**FIGURE 7:**
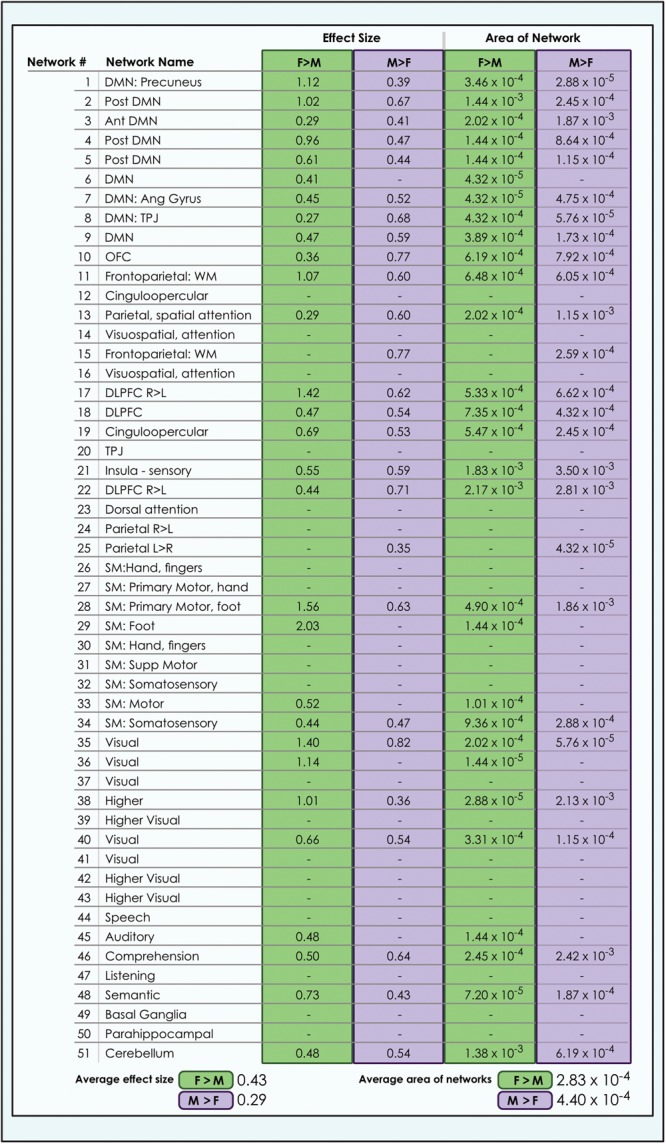
Comparison of size of sex-related effects in intrinsic networks and proportion of network affected by dimorphism in a 51-network model of brain function in 670 neurotypical young adults. The effect of sex and proportion of network affected is shown for M > F and F > M effects in a 51-network model of functional brain networks (α < 0.01, corrected for multiple comparisons and false discovery rate). The fraction of each network map with sex-related effects was computed as a percentage of all voxels analyzed from resting-state functional MRI. Average area is calculated as the arithmetic mean of the fractions pertaining to each individual network. L, Left; R, Right; Post, Posterior; Ant, Anterior; SM, Sensorimotor; Supp, Supplementary; DLPFC, Dorsolateral Prefrontal Cortex; TPJ, Temporoparietal Junction; WM, Working Memory; OFC, Orbitofrontal; DMN, Default Mode Network; Ang Gyrus, Angular Gyrus. Network labels correspond with numbers and attributions for 3-dimensional maps available in Neurovault.

The size of sex-related effects varied across individual INs, as did the spatial area of each network that displayed dimorphism. As may be appreciated in Figure 5, some smaller-sized effects such as those in the anterior DMN or supplementary motor INs nonetheless occupied relatively larger proportions of the networks in question. This was also observed in the 8- and 51-network models (Figure 6, 7). Within each network grouping there were few discernible patterns. For example, within the DMN group, some subnetworks displayed larger F > M effects while others had larger M > F effects. Exceptions were the sensorimotor and language groups where, as model order increased, there was a trend toward most effect sizes being larger in females.

We also observed that M > F effects consistently occupied a larger average proportion of network territory than F > M. This phenomenon was replicated across all models in both the original and motion-matched samples (Figure 5–7 and Supplementary Tables 3–5). The disparity between the network area affected by differences between M > F and F > M increased as model order increased, being largest in the 51-network model. In contrast, average effect size was greater in F > M vs. M > F in all 3 models in the motion-matched sample, and in the 24- and 51-network models in the original sample. The percentage of each network’s area displaying dimorphic effects also varied among models. On average, sex-related differences were present in ∼1–2% of total network area in the 8- and 24-network models and ∼0.5% of total network area in the 51-network model (Figure 6, 7 and Supplementary Tables 3–5).

### Locations Exhibiting Dimorphic Effects Were Concentrated in Default Mode and Task-Positive Control Systems and the Cerebellum

**FIGURE 8:**
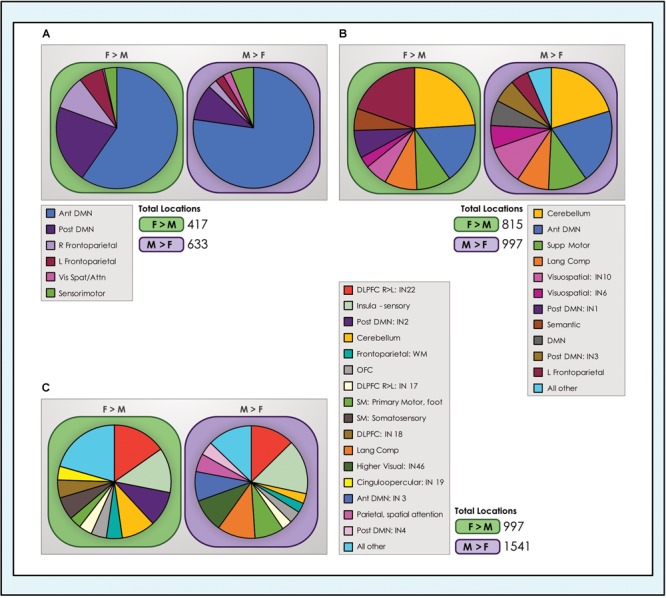
Number of dimorphic locations in intrinsic networks. The number of unique dimorphic locations (voxels) in each intrinsic network is shown for each of the **(A)** 8-network; **(B)** 24-network and **(C)** 51-network whole-brain models of brain function in neurotypical young adults. For clarity, networks with fewer than 25 and 40 dimorphic locations in the 24- and 51-network models respectively have been grouped into ‘all other’ categories. Vis spat/attn, Visuospatial/Attention; L, Left; R, Right; Post, Posterior; Ant, Anterior; Lang Comp, Language Comprehension; SM, Sensorimotor; Supp, Supplementary; DLPFC, Dorsolateral Prefrontal Cortex; TPJ, Temporoparietal Junction; WM, Working Memory; OFC, Orbitofrontal; DMN, Default Mode Network.

We computed the proportion of total locations with dimorphic effects accounted for by each network (Figure 8). Comparing across network types revealed that the preponderance of locations with sex-related differences were in control systems (default mode and task-positive INs) and the cerebellum, again regardless of model order. In the 8- and 24-network models, the anterior subnetwork of the DMN system and the left fronto-parietal network were particularly prominent. In the 51-network model, with closer delineation of subnetworks, INs anchored in the dorsolateral prefrontal cortex (IN22, a right-lateralized IN) and insula were highlighted. Interestingly, as the single cinguloopercular network observed in the 24-network model split into two INs in the 51-network model - one with more cingulate involvement and the other an insula-dominated network associated with sensory function - it was the latter that continued to exhibit sex-related differences. Other networks related to language (comprehension and semantic) and sensorimotor function also represented a meaningful share of dimorphic locations. These trends were replicated in the motion-matched sample (Supplementary Table 7), where the number of dimorphic locations increased. Typically, ∼400–600 more locations exhibited significant sex-related effects in each of the motion-matched models than in the original sample.

### The Footprint of Sex-Related Effects Across Neurocognitive Functions Was Consistently Larger in Males Than Females

**FIGURE 9:**
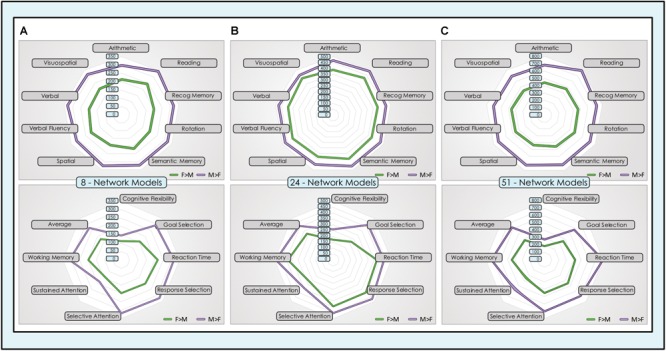
Dimorphic locations in intrinsic networks mapped to neurocognitive functions. Unique locations (voxels) with significant sex-related effects were mapped to 17 neurocognitive association test maps of brain function, derived from meta-analysis of task fMRI experiments. The top group of 9 neurocognitive functions was chosen for their prior evidence of differential performance in males and females in psychological experiments. The lower group of 8 cognitive control functions was chosen for comparison purposes. The absolute number of unique locations with dimorphic effects in all intrinsic networks concatenated across **(A)** 8-network model, **(B)** 24-network model and **(C)** 51-network model is displayed for F > M (green) and M > F (purple) effects.

We computed the spatial overlap between unique locations with significant dimorphic effects (concatenated across all networks) and association test maps for individual neurocognitive functions that have been most frequently associated with sex-related differences in task performance and in a set of cognitive control functions. In both sets of functions, the sex-related neurocognitive footprint was consistently and substantially larger in males than females, across all tasks and model orders (Figure 9). In the core set of functions most often associated with sex-related differences in neurocognitive performance, males showed 40–70% more locations in the 8-network model, 8–23% more locations in the 24-network model and 50–70% more locations in the 51-network model, averaging 55, 18, and 58% respectively. This spread was largest in the association test map associated with ‘rotation’ in the 8- and 24-network models, and ‘reading’ in the 51-network model. In the comparison set of functions less consistently associated in the behavioral literature with sex-related differences in performance, males showed 30–88% more locations in the 8-network model, 15–92% in the 24-network model and 51–90% in the 51-network model, averaging 53, 35, and 62% respectively. Across all models, the largest absolute number of dimorphic locations was consistently in ‘reading,’ in both sexes, with the exception of the 51-network model in the motion-matched sample where ‘reading’ was second behind ‘recognition memory.’

These findings were replicated in the motion-matched sample, where the number of unique dimorphic locations overlapping neurocognitive association test maps was increased. Typically, ∼200 more overlapping dimorphic locations overlapped neurocognitive maps in the 8- and 24-network models, and ∼400 more in the 51-network model (Supplementary Table 7). Of note, in the motion-matched sample there were three exceptions to our otherwise consistent findings, in the ‘spatial’ and ‘sustained attention’ maps in the 8-network model and the ‘visuospatial’ map in the 24-network model. Here, the number of unique M > F locations was slightly smaller than F > M.

### Results Summary

• Sexual dimorphism was present in the majority of intrinsic functional networks in a large group of neurotypical young adults, regardless of model order.
• The proportion of intrinsic networks with significant sex-related differences in activity ranged from 60 to 80% of all networks tested over 3 models of whole brain function.
• Significant sex-related effects were present in all sub-networks of the default mode network group in all 3 models. This contrasted with other network groupings (e.g., visual or sensorimotor networks) where sex-related effects were less pervasive
• On average, the effect size of significant sex-related differences in intrinsic network maps was larger in females, but occupied a greater spatial proportion of the network in males. The size of sex-related effects and proportion of network affected did not appear to be related.
• Control networks (default mode and task-positive intrinsic networks) and the cerebellar networks contained the largest numbers of individual brain locations (voxels) with significant sex-related differences in activity
• While our major results were replicated in the motion-matched sample, we observed a greater number of individual brain locations with significant sex-related differences in the smaller motion-matched sample of 535 subjects vs. the sample of 670 subjects matched for age and sex, but not head motion.

## Discussion

### Multilevel Modeling of Intrinsic Functional Brain Networks Revealed That the Majority Exhibit a Mosaic of Sex-Related Differences in Young Adults

A key finding of our study was that most intrinsic networks exhibit significant sex-related effects, with both F > M and M > F effects usually found within the same IN. This pervasive sexual dimorphism is present regardless of model order, appearing in each of the 8-, 24-, and 51-network models, and before or after motion-matching. This accords with previous work using ICA in rsfMRI in a 28-network model in 603 subjects (80% aged 13–30) by Allen et al. (2011) that were almost balanced for age and sex but not matched for motion. Using a different methodology with a grid-based schema and *a posteriori* assignment of nodes to network identifications in youth (average age 15 years), dimorphism was also detected by another group in a majority of intrinsic networks (Satterthwaite et al., 2015). We provide a significant contribution to previous work by performing functional parcellations with increasing model orders, determining that this phenomenon is present regardless of how refined a functional parcellation is constructed, and validating this in a motion-matched sample. These findings suggest that sex is an important biological variable in analyses of brain function in neurotypical adults, and that most networks do contain a mosaic of sex-related differences, with both M > F and F > M effects present within individual networks. Therefore, our work supports the inclusion of sex as an important biological and analytic variable in studies of intrinsic brain function using rsfMRI.

### Control Network Systems Are Most Influenced by Dimorphic Effects

Our analysis enlarges current understanding of the role of sex in functional brain networks by providing a detailed picture of dimorphism in individual network types. While we detected dimorphism in networks of all types, it was most prominent in control network systems, i.e., task-positive control networks associated with neurocognitive functions such as working memory and cognitive control (though not attention *per se*), and in the default mode networks. In the latter, we detected dimorphism across every sub-network, even as the overall default mode system split into increasingly smaller networks in the 51-network model, with some indication that posterior components of the system had larger effect sizes. Control networks – especially the DMN – dominated the spatial extent of sex-related effects, consistently occupying the lion’s share (>50%) of dimorphic locations in all models. The cerebellum was also highlighted. Our results suggest that the function of control networks and the cerebellum, especially the default mode system, may be most strongly influenced by sex in comparison to other network types. Our work therefore disagrees with smaller studies that failed to detect sex-related differences in default mode and control network function (Weissman-Fogel et al., 2010) but is congruent with others that have found sex-related differences in these networks (Biswal et al., 2010; Zuo et al., 2010). It is an intriguing finding since default mode intrinsic networks are prominently implicated in a wide variety of human neurological and neuropsychiatric disorders that have sex-related differences in incidence, prevalence and severity and have been associated with differences in cognitive control function (Mohan et al., 2016). Earlier work using task-based fMRI demonstrated that control networks including the default mode system are associated with general-purpose brain state control activities such as task initiation, maintenance and switching (Dosenbach et al., 2006, 2007), though sex-related differences were not examined. More recent studies using task-based fMRI suggest that sex-related differences do exist in task-control and default mode network function that may be specific to the task being performed and mediate clinical presentations (Dumais et al., 2018; McCarthy et al., 2019). We note that our estimates of total dimorphic functional locations across the brain varied from ∼1–2500 in the original sample to ∼2–4000 in the motion-matched sample, representing a range of ∼0.5–2% of total locations (voxels) surveyed from whole brain imaging. This is concordant with previous estimates in younger subjects using a grid-based method by Sattherthwaite et al. (2015), who found significant sex-related differences in ∼2% of nodes and ∼0.5% of edges. Thus, it appears likely that functional differences attributable to sex are present in a relatively small spatial proportion of the brain regardless of the analytic methodology applied. Similarly, a relatively small proportion of each network displays dimorphism, on average ∼0.5–1.5% of the total area of each network. Taken together, our results juxtaposed with prior studies suggest that while sex-related differences affect a relatively small spatial proportion of intrinsic network function, they may nonetheless significantly modulate information processing and behaviors, particularly those influenced by control networks and the cerebellum. Further studies will be required to test this hypothesis.

### The Neurocognitive Footprint of Intrinsic Neural Correlates Is Much Larger in Males

A remarkable finding in our work was that the neurocognitive footprint of sex-related effects was consistently larger in all models and all cognitive functions in males. The sole three exceptions were in the spatial and sustained attention maps in the 8-network model and the visuospatial map in the 24-network model in the motion-matched sample. This phenomenon was present across the range of cognitive functions we surveyed, including tasks previously associated with superior male (e.g., mental rotation) and female (e.g., reading) performance and also in a set of cognitive control functions where there has been much less consistent evidence of sex-related differences in performance. While our analytic strategy does not impute causation, this finding does suggest that cognitive task performance is not simply associated with the spatial extent of dimorphism in intrinsic networks, since the larger footprint of M > F effects was present in cognitive functions where both superior and inferior performance has been observed in males and in a range of cognitive control functions where sex-related performance differences are less robustly found. Rather, it may link to prior work suggesting males and females recruit different brain regions to accomplish similar tasks, which would also be consistent with our finding of a mosaic of M > F and F > M effects within the same networks. For example, Hugdahl et al. (2006) found that men activated the superior parietal lobule during a task of mental rotation, whereas women preferentially employed the inferior frontal. Other tasks with good evidence of sex-related differences associated with specific neural correlates include emotional face processing (Fusar-Poli et al., 2009) and emotional memory (Cahill, 2003). Our work extends these observations by revealing that the spatial extent of sex-sensitive cognitive maps is generally simply greater in males than females and involves more unique individual locations in intrinsic functional networks. In turn, this may link to the increased prevalence of neuropsychiatric disorders that involve cognitive disturbance in males (e.g., autism, schizophrenia, ADHD) vs. those that tend to have fewer cognitive impacts in females (e.g., depression, anxiety). For example, in autism a hypothesis has been advanced that male sex may confer vulnerability, or female sex protective effects, operating at the genetic and/or neural levels. An interesting comparison can be made with our prior work in ADHD, a developmental disorder with a much higher reported incidence in males, where we surveyed a similar set of cognitive control functions and found that the neurocognitive footprint across these functions was much larger in youth with ADHD vs. neurotypical youth (de Lacy et al., 2018). More broadly, our results suggest that sex-related differences in cognitive performance may have intrinsic neural correlates. For example, it is striking that we consistently found the greatest overlap between sex-sensitive locations across intrinsic networks and a neurocognitive map was with the ‘reading’ map, since reading is the task that has perhaps most robustly been determined to have sex-related performance differentials from early ages. While much work remains to be done regarding the relationship between sex/gender and neural function, and also the observed sex-related differences in both neural structure and function and human behavior (Grabowska, 2017), our results suggest that the link between sex-related differences in neural network function and cognitive task performance is not a simple quantitative relationship.

### The Effect Size of Sex, Number of Dimorphic Locations and Footprint Across Neurocognitive Functions Increased in More Refined Functional Parcellations

An important result of our study, which provides the first whole-brain surveys of dimorphism with simultaneous multilevel modeling of brain functional networks, is that sex becomes a more influential variable as increasingly detailed functional parcellations are formulated. Previous work suggests that higher model orders constructed using ICA produce increasingly refined networks and it is also reasonable to suppose that as model order increases, larger networks split into subnetworks with more closely delineated functional associations. For example, work by Andrews-Hanna et al. (2010) demonstrated that the DMN, often considered as a single network in earlier studies, in fact consists of separable sub-networks with somewhat dissociable cognitive functions. More broadly, as research methods become more advanced it is becoming increasingly popular in rsfMRI studies to construct sophisticated models of brain function containing many intrinsic networks. As we constructed more refined parcellations, the average effect size, number of dimorphic locations, and their neurocognitive footprints all increased, highlighting the particular importance of incorporating sex as a variable in more detailed models of brain function, with implications for analytic strategies and modeling. Since there is currently no principled way of determining the number of networks in human brain functional data, model order is a parameterized choice by the investigator. Our analysis suggests that the statistical significance of sex varies according to the model order chosen. While sex is associated with significant differences in the function of most intrinsic networks, its influence appears more profound as model order increases. This may also help explain disparities in prior studies which have given rise to disputes regarding the significance of sex to differences in brain function.

### Sex-Related Differences in Head Motion May Also Exist

While in general our effects were replicated in a motion-matched sample, motion may still play an important role and should always be carefully considered in fMRI studies. We found a significant difference in head motion as measured using DVARS between males and females after matching for age and sex in a sample of 670 neurotypical young adults. In the foundational dataset used for the present study, where imaging is available from a larger sample of 1570 young adults (not matched for age or sex), this was also the case (*p* = 9.365 × 10^-18^). In both cases higher levels of head motion obtained in males, particularly younger adult subjects. It is also notable that historically, higher levels of head motion have been detected in subjects with conditions such as autism and ADHD, which are more common in males, as well as in younger subjects more generally. Indeed, while efforts are increasingly devoted to identifying sources of disparity in head motion in brain imaging research, and eliminating and controlling for the effects of motion, the relationship between sex and head motion has not been extensively explored. While further studies will inform the generalizability of our findings, the current study suggests the relationship between sex and motion should be carefully accounted for in functional brain imaging studies.

### Limitations

Limitations exist in the present study. Firstly, IQ scores derived from the Shipley-Hartford Age-Corrected T-scores were provided in the original dataset and used in the current analysis, that are estimates of IQ rather than the result of full IQ testing, and as may be seen in Table 1, the average estimated IQ of this sample is above the population average. Secondly, we did not attempt to control for potential effects of hemodynamic lag on the BOLD time series. Thirdly, association test maps were utilized that represent the results of meta-analyses. While these provide the benefit of increased power, the search terms we used may be inexact or underspecified. For example, we used the term ‘mental rotation’ but the Neurosynth database at the time of our analysis did not provide the capability to specify 2- vs. 3-dimensional mental rotation. In our models of brain functional networks, we provide 3 model orders for comparison purposes, but note that the 8-network model is a relatively low model order for ICA, and did not break out certain networks or regions such as subcortical components. Moreover, while recent studies indicate that the cerebellum may be fractionated into multiple sub-networks (Buckner et al., 2011; Riedel et al., 2015) we include only a single cerebellar network in our 24- and 51-network models. Finally, we did not map all possible neurocognitive functions, but rather selected functions based on a review of the existing literature and a set of cognitive control functions selected as a comparator group. Thus, it is possible that we have not included other functions that may display sexual dimorphism.

## Conclusion

Using a multilevel modeling strategy to survey sex-related differences in intrinsic functional networks in increasing model orders of 8-, 24-, and 51-network models derived from whole-brain imaging, we identified a mosaic of sex-related effects in the majority of networks, affecting in total a small proportion of ∼0.5–2% of all brain locations. Dimorphism proved most prominent in the control networks and cerebellum, being particularly pervasive in the DMN system, with a much larger neurocognitive footprint in males. We conclude that modeling sex as a biological variable and as a covariate in analyses of human brain function using rsfMRI is required, and that high-order models which include a greater number of networks and/or more detailed functional parcellations are likely to be even more sensitive to sex-related differences. Our results extend prior investigations using task fMRI to provide additional support derived from intrinsic brain function measured in the resting state to suggest that males and females may recruit different spatial locations and proportions of brain networks to perform similar neurocognitive tasks. We highlight that our results pertain to brain function in a single, albeit comparatively large, cross-sectional sample of young adults. No conclusions can be drawn as to the causes or dynamic evolution of observed sex-related network differences in this group. The development of neural functional connectivity is a dynamic process with considerable reorganization and resculpting observed during maturation through young adulthood (Power et al., 2010). Many biological, behavioral and environmental factors are known to impact brain function and likely influence differences between groups, including sex-related differences. There is considerable individual variation and sex-related differences may exist on a spectrum. Further studies positioned within this framework will help disambiguate the origins and evolution of observed functional dimorphism in young adult brain networks and the drivers of this important phenomenon (Fine, 2014).

## Ethics Statement

The present study uses secondary data and was deemed not human subjects research by the University of Washington Institutional Review Board.

## Author Contributions

NdL designed the study, performed the analysis, and wrote the manuscript. VC helped design the study and contributed to writing the manuscript. JK and EM contributed to study design and writing the manuscript.

## Conflict of Interest Statement

The authors declare that the research was conducted in the absence of any commercial or financial relationships that could be construed as a potential conflict of interest.

## Footnotes

1 https://www.neuroinfo.org/gsp/

2 https://static1.squarespace.com/static/5b58b6da7106992fb15f7d50/t/5b68650d8a922db3bb807a90/1533568270847/GSP_README_140630.pdf

3 http://www.fmriconsulting.com/brodmann/Interact.html

4 http://neurosynth.org/

## References

[B1] Allen E. A. Erhardt E. B. Damaraju E. Gruner W. Segall J. M. Silva R. F. (2011). A baseline for the multivariate comparison of resting-state networks. *Front. Syst. Neurosci.* 5:2 10.3389/fnsys.2011.00002 PMC3051178 21442040

[B2] Allen E. A. Erhardt E. B. Wei Y. Eichele T. Calhoun V. D. (2012). Capturing inter-subject variability with group independent component analysis of fMRI data: a simulation study. *Neuroimage* 59 4141–4159. 10.1016/j.neuroimage.2011.10.010 22019879 PMC3690335

[B3] Andrews-Hanna J. R. Reidler J. S. Sepulcre J. Poulin R. Buckner R. L. (2010). Functional-anatomic fractionation of the brain’s default network. *Neuron* 65 550–562. 10.1016/j.neuron.2010.02.005 20188659 PMC2848443

[B4] Biswal B. B. Mennes M. Zuo X. N. Gohel S. Kelly C. Smith S. M. (2010). Toward discovery science of human brain function. *Proc. Natl. Acad. Sci. U.S.A.* 107 4734–4739. 10.1073/pnas.0911855107 20176931 PMC2842060

[B5] Buckner R. L. Krienen F. M. Castellanos A. Diaz J. C. Yeo B. T. (2011). The organization of the human cerebellum estimated by intrinsic functional connectivity. *J. Neurophysiol.* 106 2322–2345. 10.1152/jn.00339.2011 21795627 PMC3214121

[B6] Cahill L. (2003). Sex-related influences on the neurobiology of emotionally influenced memory. *Ann. N.Y. Acad. Sci.* 985 163–173. 10.1111/j.1749-6632.2003.tb07080.x 12724157

[B7] Calhoun V. D. Adali T. (2012). Multisubject independent component analysis of fMRI: a decade of intrinsic networks, default mode, and neurodiagnostic discovery. *IEEE Rev. Biomed. Eng.* 5 60–73. 10.1109/RBME.2012.2211076 23231989 PMC4433055

[B8] Calhoun V. D. Adali T. Pearlson G. D. Pekar J. J. (2001). A method for making group inferences from functional MRI data using independent component analysis. *Hum. Brain Mapp.* 14 140–151. 10.1002/hbm.1048 11559959 PMC6871952

[B9] Christodoulou A. G. Bauer T. E. Kiehl K. A. Feldstein Ewing S. W. Bryan A. D. Calhoun V. D. (2013). A quality control method for detecting and suppressing uncorrected residual motion in fMRI studies. *Magn. Reson. Imaging* 31 707–717. 10.1016/j.mri.2012.11.007 23290482 PMC3648631

[B10] de Lacy N. Doherty D. King B. H. Rachakonda S. Calhoun V. D. (2017). Disruption to control network function correlates with altered dynamic connectivity in the wider autism spectrum. *Neuroimage Clin.* 15 513–524. 10.1016/j.nicl.2017.05.024 28652966 PMC5473646

[B11] de Lacy N. Kodish I. Rachakonda S. Calhoun V. D. (2018). Novel in silico multivariate mapping of intrinsic and anticorrelated connectivity to neurocognitive functional maps supports the maturational hypothesis of ADHD. *Hum Brain Mapp.* 39 3449–3467. 10.1002/hbm.24187 29682852 PMC6045974

[B12] Dosenbach N. U. Fair D. A. Miezin F. M. Cohen A. L. Wenger K. K. Dosenbach R. A. (2007). Distinct brain networks for adaptive and stable task control in humans. *Proc. Natl. Acad. Sci. U.S.A.* 104 11073–11078. 10.1073/pnas.0704320104 17576922 PMC1904171

[B13] Dosenbach N. U. Visscher K. M. Palmer E. D. Miezin F. M. Wenger K. K. Kang H. C. (2006). A core system for the implementation of task sets. *Neuron* 50 799–812. 10.1016/j.neuron.2006.04.031 16731517 PMC3621133

[B14] Du Y. Allen E. A. He H. Sui J. Wu L. Calhoun V. D. (2016). Artifact removal in the context of group ICA: A comparison of single-subject and group approaches. *Hum. Brain Mapp.* 37 1005–1025. 10.1002/hbm.23086 26859308 PMC5784424

[B15] Dumais K. M. Chernyak S. Nickerson L. D. Janes A. C. (2018). Sex differences in default mode and dorsal attention network engagement. *PLoS One* 13:e0199049. 10.1371/journal.pone.0199049 29902249 PMC6002059

[B16] Erhardt E. B. Rachakonda S. Bedrick E. J. Allen E. A. Adali T. Calhoun V. D. (2011). Comparison of multi-subject ICA methods for analysis of fMRI data. *Hum. Brain Mapp.* 32 2075–2095. 10.1002/hbm.21170 21162045 PMC3117074

[B17] Fine C. (2014). Neuroscience. His brain, her brain? *Science* 346 915–916. 10.1126/science.1262061 25414288

[B18] Fox M. D. Snyder A. Z. Vincent J. L. Corbetta M. Van Essen D. C. Raichle M. E. (2005). The human brain is intrinsically organized into dynamic, anticorrelated functional networks. *Proc. Natl. Acad. Sci. U.S.A.* 102 9673–9678. 10.1073/pnas.0504136102 15976020 PMC1157105

[B19] Fusar-Poli P. Placentino A. Carletti F. Landi P. Allen P. Surguladze S. (2009). Functional atlas of emotional faces processing: a voxel-based meta-analysis of 105 functional magnetic resonance imaging studies. *J. Psychiatry Neurosci.* 34 418–432. 19949718 PMC2783433

[B20] Gong G. He Y. Evans A. C. (2011). Brain connectivity: gender makes a difference. *Neuroscientist* 17 575–591. 10.1177/1073858410386492 21527724

[B21] Grabowska A. (2017). Sex on the brain: are gender-dependent structural and functional differences associated with behavior? *J. Neurosci. Res.* 95 200–212. 10.1002/jnr.23953 27870447

[B22] Guadalupe T. Mathias S. R. vanErp T. G. M. Whelan C. D. Zwiers M. P. Abe Y. (2017). Human subcortical brain asymmetries in 15,847 people worldwide reveal effects of age and sex. *Brain Imaging Behav.* 11 1497–1514. 10.1007/s11682-016-9629-z 27738994 PMC5540813

[B23] Gur R. C. Alsop D. Glahn D. Petty R. Swanson C. L. Maldjian J. A. (2000). An fMRI study of sex differences in regional activation to a verbal and a spatial task. *Brain Lang.* 74 157–170. 10.1006/brln.2000.2325 10950912

[B24] Gur R. C. Turetsky B. I. Matsui M. Yan M. Bilker W. Hughett P. (1999). Sex differences in brain gray and white matter in healthy young adults: correlations with cognitive performance. *J. Neurosci.* 19 4065–4072. 10.1523/JNEUROSCI.19-10-04065.1999 10234034 PMC6782697

[B25] Himberg J. Hyvarinen A. Esposito F. (2004). Validating the independent components of neuroimaging time series via clustering and visualization. *Neuroimage* 22 1214–1222. 10.1016/j.neuroimage.2004.03.027 15219593

[B26] Holmes A. J. Hollinshead M. O. O’Keefe T. M. Petrov V. I. Fariello G. R. Wald L. L. (2015). Brain genomics superstruct project initial data release with structural, functional, and behavioral measures. *Sci. Data* 2:150031. 10.1038/sdata.2015.31 26175908 PMC4493828

[B27] Hugdahl K. Thomsen T. Ersland L. (2006). Sex differences in visuo-spatial processing: an fMRI study of mental rotation. *Neuropsychologia* 44 1575–1583. 10.1016/j.neuropsychologia.2006.01.026 16678867

[B28] Im K. Lee J. M. Lee J. Shin Y. W. Kim I. Y. Kwon J. S. (2006). Gender difference analysis of cortical thickness in healthy young adults with surface-based methods. *Neuroimage* 31 31–38. 10.1016/j.neuroimage.2005.11.042 16426865

[B29] Jamadar S. D. Sforazzini F. Raniga P. Ferris N. J. Paton B. Bailey M. J. (2018). Sexual dimorphism of resting-state network connectivity in healthy ageing. *J. Gerontol. B Psychol. Sci. Soc. Sci.* 10.1093/geronb/gby004 [Epub ahead of print]. 29471348 PMC6748717

[B30] Laird A. R. Fox P. M. Eickhoff S. B. Turner J. A. Ray K. L. McKay D. R. (2011). Behavioral interpretations of intrinsic connectivity networks. *J. Cogn. Neurosci.* 23 4022–4037. 10.1162/jocn-a-00077 21671731 PMC3690655

[B31] Li R. Singh M. (2014). Sex differences in cognitive impairment and Alzheimer’s disease. *Front. Neuroendocrinol.* 35 385–403. 10.1016/j.yfrne.2014.01.002 24434111 PMC4087048

[B32] Li Y. O. Adali T. Calhoun V. D. (2007). Estimating the number of independent components for functional magnetic resonance imaging data. *Hum. Brain Mapp.* 28 1251–1266. 10.1002/hbm.20359 17274023 PMC6871474

[B33] McCarthy J. M. Dumais K. M. Zegel M. Pizzagalli D. A. Olson D. P. Moran L. V. (2019). Sex differences in tobacco smokers: Executive control network and frontostriatal connectivity. *Drug Alcohol Depend* 195 59–65. 10.1016/j.drugalcdep.2018.11.023 30592997 PMC6625360

[B34] Miller D. I. Halpern D. F. (2014). The new science of cognitive sex differences. *Trends Cogn. Sci.* 18 37–45. 10.1016/j.tics.2013.10.011 24246136

[B35] Mohan A. Roberto A. J. Mohan A. Lorenzo A. Jones K. Carney M. J. (2016). The significance of the default mode network (DMN) in neurological and neuropsychiatric disorders: a review. *Yale J. Biol. Med.* 89 49–57. 27505016 PMC4797836

[B36] Mutlu A. K. Schneider M. Debbane M. Badoud D. Eliez S. Schaer M. (2013). Sex differences in thickness, and folding developments throughout the cortex. *Neuroimage* 82 200–207. 10.1016/j.neuroimage.2013.05.076 23721724

[B37] Podcasy J. L. Epperson C. N. (2016). Considering sex and gender in Alzheimer disease and other dementias. *Dial. Clin. Neurosci.* 18 437–446. 10.31887/DCNS.2016.18.4/cepperson PMC5286729 28179815

[B38] Power J. D. Fair D. A. Schlaggar B. L. Petersen S. E. (2010). The development of human functional brain networks. *Neuron* 67 735–748. 10.1016/j.neuron.2010.08.017 20826306 PMC2941973

[B39] Power J. D. Mitra A. Laumann T. O. Snyder A. Z. Schlaggar B. L. Petersen S. E. (2014). Methods to detect, characterize, and remove motion artifact in resting state fMRI. *Neuroimage* 84 320–341. 10.1016/j.neuroimage.2013.08.048 23994314 PMC3849338

[B40] Rashid B. Damaraju E. Pearlson G. D. Calhoun V. D. (2014). Dynamic connectivity states estimated from resting fMRI Identify differences among Schizophrenia, bipolar disorder, and healthy control subjects. *Front. Hum. Neurosci.* 8:897. 10.3389/fnhum.2014.00897 25426048 PMC4224100

[B41] Riedel M. C. Ray K. L. Dick A. S. Sutherland M. T. Hernandez Z. Fox P. M. (2015). Meta-analytic connectivity and behavioral parcellation of the human cerebellum. *Neuroimage* 117 327–342. 10.1016/j.neuroimage.2015.05.008 25998956 PMC4512917

[B42] Ritchie S. J. Cox S. R. Shen X. Lombardo M. V. Reus L. M. Alloza C. (2018). Sex differences in the adult human brain: evidence from 5216 UK biobank participants. *Cereb. Cortex* 28 2959–2975. 10.1093/cercor/bhy109 29771288 PMC6041980

[B43] Sacher J. Neumann J. Okon-Singer H. Gotowiec S. Villringer A. (2013). Sexual dimorphism in the human brain: evidence from neuroimaging. *Magn. Reson. Imaging* 31 366–375. 10.1016/j.mri.2012.06.007 22921939

[B44] Satterthwaite T. D. Wolf D. H. Roalf D. R. Ruparel K. Erus G. Vandekar S. (2015). Linked sex differences in cognition and functional connectivity in youth. *Cereb. Cortex* 25 2383–2394. 10.1093/cercor/bhu036 24646613 PMC4537416

[B45] Seeley W. W. Menon V. Schatzberg A. F. Keller J. Glover G. H. Kenna H. (2007). Dissociable intrinsic connectivity networks for salience processing and executive control. *J. Neurosci.* 27 2349–2356. 10.1523/JNEUROSCI.5587-06.2007 17329432 PMC2680293

[B46] Smith S. M. Fox P. T. Miller K. L. Glahn D. C. Fox P. M. Mackay C. E. (2009). Correspondence of the brain’s functional architecture during activation and rest. *Proc. Natl. Acad. Sci. U.S.A.* 106 13040–13045. 10.1073/pnas.0905267106 19620724 PMC2722273

[B47] Spreng R. N. Sepulcre J. Turner G. R. Stevens W. D. Schacter D. L. (2013). Intrinsic architecture underlying the relations among the default, dorsal attention, and frontoparietal control networks of the human brain. *J. Cogn. Neurosci.* 25 74–86. 10.1162/jocn-a-00281 22905821 PMC3816715

[B48] Stoet G. Geary D. C. (2013). Sex differences in mathematics and reading achievement are inversely related: within- and across-nation assessment of 10 years of PISA data. *PLoS One* 8:e57988. 10.1371/journal.pone.0057988 23516422 PMC3596327

[B49] Van Dijk K. R. Hedden T. Venkataraman A. Evans K. C. Lazar S. W. Buckner R. L. (2010). Intrinsic functional connectivity as a tool for human connectomics: theory, properties, and optimization. *J. Neurophysiol.* 103 297–321. 10.1152/jn.00783.2009 19889849 PMC2807224

[B50] Vernet M. Quentin R. Chanes L. Mitsumasu A. Valero-Cabre A. (2014). Frontal eye field, where art thou? Anatomy, function, and non-invasive manipulation of frontal regions involved in eye movements and associated cognitive operations. *Front. Integr. Neurosci.* 8:66. 10.3389/fnint.2014.00066 25202241 PMC4141567

[B51] Voyer D. Voyer S. Bryden M. P. (1995). Magnitude of sex differences in spatial abilities: a meta-analysis and consideration of critical variables. *Psychol. Bull.* 117 250–270. 10.1037/0033-2909.117.2.250 7724690

[B52] Weissman-Fogel I. Moayedi M. Taylor K. S. Pope G. Davis K. D. (2010). Cognitive and default-mode resting state networks: do male and female brains ”rest” differently? *Hum. Brain Mapp.* 31 1713–1726. 10.1002/hbm.20968 20725910 PMC6870948

[B53] Yang J. Lee J. (2018). Different aberrant mentalizing networks in males and females with autism spectrum disorders: evidence from resting-state functional magnetic resonance imaging. *Autism* 22 134–148. 10.1177/1362361316667056 29490484

[B54] Yarkoni T. Poldrack R. A. Nichols T. E. Van Essen D. C. Wager T. D. (2011). Large-scale automated synthesis of human functional neuroimaging data. *Nat. Methods* 8 665–670. 10.1038/nmeth.1635 21706013 PMC3146590

[B55] Zuo X. N. Kelly C. Di Martino A. Mennes M. Margulies D. S. Bangaru S. (2010). Growing together and growing apart: regional and sex differences in the lifespan developmental trajectories of functional homotopy. *J. Neurosci.* 30 15034–15043. 10.1523/JNEUROSCI.2612-10.2010 21068309 PMC2997358

