# Supplementary material for: Multilevel Mapping of Sexual Dimorphism in Intrinsic Functional Brain Networks
Source: Front Neurosci. 2019 Apr 5;13:332. doi: 10.3389/fnins.2019.00332 (PMC6460937; doi:10.3389/fnins.2019.00332)
Supplement: Supplementary file 3 [file Table_3.DOCX]

**Supplementary Table 3: Effect size and area of network affected in 8-network model**

|  |  | **Effect Size** | | **Area of Network** | |
| --- | --- | --- | --- | --- | --- |
| **Network #** | **Network Name** | **F>M** | **M>F** | **F>M** | **M>F** |
| 1 | Anterior DMN | 0.31 | 0.41 | 7.22 x 10^-3^ | 1.02 x 10^-2^ |
| 2 | Posterior DMN | 0.49 | 0.26 | 3.80 x 10^-3^ | 2.25 x 10^-3^ |
| 3 | R Frontoparietal | 0.41 | 0.35 | 4.03 x 10^-4^ | 2.16 x 10^-4^ |
| 4 | L Frontoparietal | 0.36 | 0.27 | 1.30 x 10^-3^ | 4.32x 10^-4^ |
| 5 | Vis Spat/Attn | 0.27 | 0.39 | 5.62 x 10^-4^ | 8.07 x 10^-4^ |
| 6 | Sensorimotor | 0.48 | 0.36 | 1.11 x 10^-3^ | 1.41 x 10^-3^ |
| 7 | Visual | - | 0.96 | - | 5.76 x 10^-5^ |
| 8 | Visual | - | - | - | - |
| **Average** |  | **0.28** | **0.37** | **1.80 x 10^-3^** | **1.92 x 10^-3^** |
