# Supplementary material for: Multilevel Mapping of Sexual Dimorphism in Intrinsic Functional Brain Networks
Source: Front Neurosci. 2019 Apr 5;13:332. doi: 10.3389/fnins.2019.00332 (PMC6460937; doi:10.3389/fnins.2019.00332)
Supplement: Supplementary file 4 [file Table_4.DOCX]

**Supplementary Table 4: Effect size and area of network affected in 24-network model**

|  |  | **Effect Size** | | **Area of Network** | |
| --- | --- | --- | --- | --- | --- |
| **Network #** | **Network Name** | **F>M** | **M>F** | **F>M** | **M>F** |
| 1 | Posterior DMN | 1.06 | 0.28 | 9.07 x 10^-4^ | 1.44 x 10^-4^ |
| 2 | Anterior DMN | 0.46 | 0.63 | 4.09 x 10^‑3^ | 4.41 x 10^-3^ |
| 3 | Posterior DMN | 0.41 | 0.69 | 2.59 x 10^-4^ | 8.64 x 10^-4^ |
| 4 | DMN | 0.24 | 0.37 | 3.02 x 10^-4^ | 2.17 x 10^-3^ |
| 5 | Orbitofrontal | 0.42 | 0.79 | 2.16 x 10^-4^ | 2.16 x 10^-4^ |
| 6 | Visuospatial | 0.48 | 0.70 | 7.20 x 10^-4^ | 1.25 x 10^-3^ |
| 7 | Prefrontal | 0.41 | 0.42 | 3.89 x 10^-4^ | 2.02 x 10^-4^ |
| 8 | Right frontoparietal | 0.55 | 0.44 | 7.35 x 10^-4^ | 2.45 x 10^-4^ |
| 9 | Left frontoparietal | 0.40 | 0.82 | 3.60 x 10^-4^ | 9.79 x 10^-4^ |
| 10 | Visuospatial | 0.76 | 0.62 | 1.4 x 10^-3^ | 2.3 x 10^-3^ |
| 11 | Cinguloopercular | 0.26 | 0.49 | 3.75 x 10^-4^ | 7.35 x 10^-4^ |
| 12 | Sensory | 0.48 | 0.50 | 5.04 x 10^-4^ | 3.16 x 10^-4^ |
| 13 | Supplementary Motor | 0.43 | 0.51 | 2.13 x 10^-3^ | 2.23 x 10^-3^ |
| 14 | Primary Motor | 1.55 | 0.51 | 4.03 x 10^-4^ | 1.01 x 10^-4^ |
| 15 | Sensorimotor:Gesture | 0.41 | 0.39 | 7.20 x 10^-4^ | 1.87 x 10^-4^ |
| 16 | Motor Control | - | 0.88 | - | 2.45 x 10^-4^ |
| 17 | Primary Visual | - | - | - | - |
| 18 | Visual | - | - | - | - |
| 19 | Visual | - | - | - | - |
| 20 | Speech | 0.92 | 0.68 | 1.58 x 10^-4^ | 2.45 x 10^-4^ |
| 21 | Auditory | 0.50 | 0.58 | 3.75 x 10^-4^ | 1.87 x 10^-4^ |
| 22 | Semantic | 0.45 | 0.47 | 1.08 x 10^-3^ | 3.02 x 10^-4^ |
| 23 | Comprehension | 0.47 | 0.61 | 2.26 x 10^-3^ | 1.84 x 10^-3^ |
| 24 | Cerebellum | 0.47 | 0.46 | 3.37 x 10^-3^ | 3.68 x 10^-3^ |
| **Average** |  | **0.44** | **0.50** | **8.66 x 10^-4^** | **9.53 x 10^-4^** |
