# Supplementary material for: Multilevel Mapping of Sexual Dimorphism in Intrinsic Functional Brain Networks
Source: Front Neurosci. 2019 Apr 5;13:332. doi: 10.3389/fnins.2019.00332 (PMC6460937; doi:10.3389/fnins.2019.00332)
Supplement: Supplementary file 5 [file Table_5.DOCX]

**Supplementary Table 5: Effect size and area of network affected in 51-network model**

|  |  | **Effect Size** | | **Area of Network** | |
| --- | --- | --- | --- | --- | --- |
| **Network #** | **Network Name** | **F>M** | **M>F** | **F>M** | **M>F** |
| 1 | DMN: Precuneus | 1.30 | 0.36 | 3.46 x 10^-4^ | 1.44 x 10^-5^ |
| 2 | Posterior DMN | 1.09 | 0.97 | 1.60 x 10^-3^ | 5.04 x 10^-4^ |
| 3 | Anterior DMN | 0.34 | 0.44 | 6.05 x 10^-4^ | 2.98 x 10^-3^ |
| 4 | Posterior DMN | 0.75 | 0.45 | 2.16 x 10^-4^ | 9.36 x 10^-4^ |
| 5 | Posterior DMN | 0.95 | 0.37 | 4.32 x 10^-4^ | 1.58 x 10^-4^ |
| 6 | DMN | 0.49 | 0.72 | 5.62 x 10^-4^ | 1.44 x 10^-4^ |
| 7 | DMN: Angular Gyrus | 0.44 | 0.56 | 2.30 x 10^-4^ | 1.34 x 10^-3^ |
| 8 | DMN: TPJ | 0.42 | 0.63 | 4.03 x 10^-4^ | 8.79 x 10^-4^ |
| 9 | DMN | 0.54 | 0.56 | 4.61 x 10^-4^ | 4.18 x 10^-4^ |
| 10 | Orbitofrontal | 0.40 | 0.83 | 7.78 x 10^-4^ | 1.22 x 10^-3^ |
| 11 | Frontoparietal: WM | 0.85 | 0.60 | 1.58 x 10^-3^ | 1.67 x 10^-3^ |
| 12 | Cinguloopercular | 0.44 | 0.67 | 1.44 x 10^-5^ | 2.45 x 10^-4^ |
| 13 | Parietal: Spatial Attention | 0.42 | 0.66 | 8.53 x 10^-4^ | 1.48 x 10^-3^ |
| 14 | Visuospatial Attention | - | - | - | - |
| 15 | Frontoparietal: WM | 0.53 | 0.87 | 8.64 x 10^-5^ | 2.88 x 10^-4^ |
| 16 | Visuospatial Attention | - | - | - | - |
| 17 | DLPFC R>L | 1.34 | 0.70 | 8.50 x 10^-4^ | 6.05 x 10^-4^ |
| 18 | DLPFC | 0.47 | 0.58 | 1.04 x 10^-3^ | 1.09 x 10^-3^ |
| 19 | Cinguloopercular | 0.75 | 0.55 | 1.30 x 10^-3^ | 2.88 x 10^-4^ |
| 20 | TPJ | 0.57 | 0.48 | 1.15 x 10^-3^ | 2.16 x 10^-4^ |
| 21 | Insula: Sensory | 0.55 | 0.64 | 3.44 x 10^-3^ | 5.20 x 10^-3^ |
| 22 | DLPFC R>L | 0.48 | 0.80 | 3.86 x 10^-3^ | 3.99 x 10^-3^ |
| 23 | Dorsal Attention | - | - | - | - |
| 24 | Parietal R>L | 0.48 | 0.59 | 1.15 x 10^-4^ | 4.32 x 10^-5^ |
| 25 | Parietal L>R | - | 0.41 | - | 2.88 x 10^-5^ |
| 26 | SM: Hand, Fingers | - | - | - | - |
| 27 | SM: Primary Motor, Hand | - | - | - | - |
| 28 | SM: Primary Motor, Foot | 1.37 | 0.66 | 7.49 x 10^-4^ | 2.20 x 10^-3^ |
| 29 | SM: Foot | 2.11 | - | 2.30 x 10^-4^ | - |
| 30 | SM: Hand, Fingers | 0.45 | - | 1.44 x 10^-4^ | - |
| 31 | SM: Supp Motor | - | - | - | - |
| 32 | SM: Somatosensory | - | - | - | - |
| 33 | SM: Motor | 0.58 | 0.55 | 1.44 x 10^-4^ | 1.44 x 10^-5^ |
| 34 | SM: Somatosensory | 0.52 | 0.47 | 1.47 x 10^-3^ | 3.74 x 10^-4^ |
| 35 | Visual | 1.44 | 1.01 | 3.74 x 10^-4^ | 1.58 x 10^-4^ |
| 36 | Visual | - | - | - | - |
| 37 | Visual | - | - | - | - |
| 38 | Higher Visual | 0.88 | 0.41 | 4.18 x 10^-4^ | 2.98 x 10^-3^ |
| 39 | Higher Visual | - | - | - | - |
| 40 | Visual | 0.77 | 0.58 | 3.74 x 10^-4^ | 1.15 x 10^-4^ |
| 41 | Visual | - | - | - | - |
| 42 | Higher Visual | - | 0.29 | - | 2.88 x 10^-5^ |
| 43 | Higher Visual | - | - | - | - |
| 44 | Speech | 1.28 | 0.44 | 4.32 x 10^-5^ | 2.88 x 10^-5^ |
| 45 | Auditory | 0.59 | 0.70 | 1.01 x 10^-4^ | 5.76 x 10^-5^ |
| 46 | Comprehension | 0.50 | 0.71 | 6.34 x 10^-4^ | 2.97 x 10^-3^ |
| 47 | Listening | - | 0.86 | - | 1.44 x 10^-5^ |
| 48 | Semantic | 0.67 | 0.50 | 2.88 x 10^-4^ | 4.90 x 10^-4^ |
| 49 | Basal Ganglia | - | - | - | - |
| 50 | Parahippocampal | - | - | - | - |
| 51 | Cerebellum | 0.51 | 0.53 | 1.74 x 10^-3^ | 8.93 x 10^-4^ |
| **Average** |  | **0.50** | **0.42** | **5.22 x 10^-4^** | **6.68 x 10^-4^** |
