# Supplementary material for: Multilevel Mapping of Sexual Dimorphism in Intrinsic Functional Brain Networks
Source: Front Neurosci. 2019 Apr 5;13:332. doi: 10.3389/fnins.2019.00332 (PMC6460937; doi:10.3389/fnins.2019.00332)
Supplement: Supplementary file 6 [file Table_6.DOCX]

**Supplementary Table 6: Comparison of network locations with dimorphic effects in motion-matched sample**

1. **8-network model**

| **Network** | **F>M** | **Percent** | **M>F** | **Percent** |
| --- | --- | --- | --- | --- |
| Anterior DMN | 502 | 50.0 | 711 | 66.3 |
| Posterior DMN | 265 | 26.4 | 156 | 14.6 |
| R Frontoparietal | 29 | 2.9 | 15 | 1.4 |
| L Frontoparietal | 90 | 9.0 | 31 | 2.9 |
| Vis Spat/Attn | 40 | 4.0 | 56 | 5.2 |
| Sensorimotor | 78 | 7.8 | 98 | 9.1 |
| Visual | - | - | 5 | 0.5 |
| Visual | - | - | - | - |
| **Total** | **1004** |  | **1072** |  |

1. **24-network model**

| **Network** | **F>M** | **Percent** | **M>F** | **Percent** |
| --- | --- | --- | --- | --- |
| Posterior DMN | 63 | 4.3 | 11 | 0.7 |
| Anterior DMN | 284 | 19.6 | 307 | 19.2 |
| Posterior DMN | 19 | 1.3 | 60 | 3.4 |
| DMN | 21 | 1.5 | 152 | 9.5 |
| OFC | 15 | 1.0 | 16 | 1.0 |
| Visuospatial | 51 | 3.5 | 87 | 5.4 |
| Prefrontal | 28 | 1.9 | 14 | 0.9 |
| R Frontoparietal | 52 | 3.6 | 17 | 1.1 |
| L Frontoparietal | 25 | 1.7 | 69 | 4.3 |
| Visuospatial | 100 | 6.9 | 162 | 10.1 |
| Cinguloopercular | 26 | 1.8 | 52 | 3.3 |
| Sensory | 35 | 2.4 | 23 | 1.4 |
| Supp Motor | 149 | 10.3 | 155 | 9.7 |
| Prim Motor | 28 | 1.9 | 8 | 0.5 |
| SM Gesture | 50 | 3.4 | 14 | 0.9 |
| Motor Cont | - | - | 18 | 1.1 |
| Prim Visual | - | - | - | - |
| Visual | - | - | - | - |
| Visual | - | - | - | - |
| Speech | 12 | 0.8 | 17 | 1.1 |
| Auditory | 26 | 1.8 | 14 | 0.9 |
| Semantic | 76 | 5.2 | 21 | 1.3 |
| Lang Comp | 158 | 10.9 | 128 | 8.0 |
| Cerebellum | 235 | 16.2 | 256 | 16.0 |
| **Total** | **1453** |  | **1601** |  |

1. **51-network model**

| **Network** | **F>M** | **Percent** | **M>F** | **Percent** |
| --- | --- | --- | --- | --- |
| DMN: Precuneus | 25 | 1.3 | 1 | <0.1 |
| Posterior DMN | 112 | 6.0 | 35 | 1.5 |
| Anterior DMN | 42 | 2.3 | 208 | 8.7 |
| Posterior DMN | 15 | 0.8 | 66 | 2.8 |
| Posterior DMN | 31 | 1.7 | 11 | 0.5 |
| DMN | 40 | 2.1 | 10 | 0.4 |
| DMN: Ang Gyrus | 16 | 0.9 | 94 | 4.0 |
| DMN: TPJ | 29 | 1.6 | 61 | 2.6 |
| DMN | 33 | 1.8 | 29 | 1.2 |
| OFC | 55 | 2.9 | 85 | 3.6 |
| Frontoparietal: WM | 111 | 5.9 | 116 | 4.9 |
| Cinguloopercular | 1 | <0.1 | 18 | 0.8 |
| Parietal: Spat Attn | 58 | 3.1 | 104 | 4.4 |
| Visuospatial, Attn | - | - | - | - |
| Frontoparietal: WM | 6 | 0.3 | 21 | 0.9 |
| Visuospatial, Attn | - | - | 1 | <0.1 |
| DLPFC R>L | 59 | 3.2 | 43 | 1.8 |
| DLPFC | 73 | 3.9 | 76 | 3.2 |
| Cinguloopercular | 90 | 4.8 | 21 | 0.9 |
| TPJ | 81 | 4.3 | 15 | 0.6 |
| Insula: sensory | 239 | 12.8 | 362 | 15.2 |
| DLPFC R>L | 268 | 14.3 | 278 | 11.7 |
| Dorsal attention | - | - | - | - |
| Parietal R>L | 9 | 0.5 | 3 | 0.1 |
| Parietal L>R | - | - | 3 | 0.1 |
| SM: Hand, fingers | - | - | - | - |
| Prim motor, hand | - | - | - | - |
| Prim motor, foot | 52 | 2.8 | 154 | 6.5 |
| SM: Foot | 17 | 0.9 | - | - |
| SM: Hand, fingers | 11 | 0.6 | - | - |
| Supp motor | - | - | - | - |
| Somatosensory | - | - | - | - |
| SM: Motor | 11 | 0.6 | 1 | <0.1 |
| Somatosensory | 103 | 5.5 | 26 | 1.1 |
| Visual | 26 | 1.4 | 12 | 0.5 |
| Visual | 1 | <0.1 | - | - |
| Visual | - | - | - | - |
| Higher visual | 30 | 1.6 | 207 | 8.7 |
| Higher visual | - | - | - | - |
| Visual | 27 | 1.4 | 8 | 0.3 |
| Visual | - | - | - | - |
| Higher visual | - | - | 3 | 0.1 |
| Higher visual | - | - | - | - |
| Speech | 4 | 0.2 | 2 | 0.1 |
| Auditory | 8 | 0.4 | 4 | 0.2 |
| Comprehension | 44 | 2.4 | 207 | 8.7 |
| Listening | 1 | 0.1 | 1 | <0.1 |
| Semantic | 20 | 1.1 | 35 | 1.5 |
| Basal Ganglia | - | - | - | - |
| Parahippocampal | - | - | - | - |
| Cerebellum | 122 | 6.5 | 62 | 2.6 |
| **Total** | **1870** |  | **2383** |  |
