# Supplementary material for: Multilevel Mapping of Sexual Dimorphism in Intrinsic Functional Brain Networks
Source: Front Neurosci. 2019 Apr 5;13:332. doi: 10.3389/fnins.2019.00332 (PMC6460937; doi:10.3389/fnins.2019.00332)
Supplement: Supplementary file 7 [file Table_7.DOCX]

**Supplementary Table 7: Number of dimorphic locations in selected neurocognitive reverse inference maps in motion-matched sample**

1. **8-network model**

| **Reverse Inference Map** | **F>M number of locations** | **M>F number of locations** |
| --- | --- | --- |
| Arithmetic | 494 | 526 |
| Reading | 573 | 582 |
| Recognition Memory | 506 | 544 |
| Rotation | 504 | 531 |
| Semantic Memory | 517 | 545 |
| Spatial | 524 | 517 |
| Verbal Fluency | 501 | 525 |
| Verbal | 489 | 525 |
| Visuospatial | 502 | 557 |
| Cognitive Flexibility | 222 | 261 |
| Goal Selection | 348 | 441 |
| Reaction Time | 520 | 532 |
| Response Selection | 502 | 556 |
| Selective Attention | 512 | 523 |
| Sustained Attention | 376 | 331 |
| Working Memory | 493 | 542 |

1. **24-network model**

| **Reverse Inference Map** | **F>M number of locations** | **M>F number of locations** |
| --- | --- | --- |
| Arithmetic | 685 | 730 |
| Reading | 747 | 775 |
| Recognition Memory | 699 | 716 |
| Rotation | 685 | 750 |
| Semantic Memory | 714 | 727 |
| Spatial | 684 | 743 |
| Verbal Fluency | 702 | 729 |
| Verbal | 723 | 734 |
| Visuospatial | 726 | 714 |
| Cognitive Flexibility | 304 | 406 |
| Goal Selection | 454 | 640 |
| Reaction Time | 675 | 705 |
| Response Selection | 706 | 715 |
| Selective Attention | 708 | 721 |
| Sustained Attention | 511 | 535 |
| Working Memory | 720 | 760 |

1. **51-network model**

| **Reverse Inference Map** | **F>M number of locations** | **M>F number of locations** |
| --- | --- | --- |
| Arithmetic | 841 | 1080 |
| Reading | 878 | 1160 |
| Recognition Memory | 892 | 1108 |
| Rotation | 857 | 1099 |
| Semantic Memory | 857 | 1083 |
| Spatial | 848 | 1086 |
| Verbal Fluency | 867 | 1108 |
| Verbal | 876 | 1122 |
| Visuospatial | 830 | 1063 |
| Cognitive Flexibility | 347 | 499 |
| Goal Selection | 669 | 898 |
| Reaction Time | 816 | 1090 |
| Response Selection | 838 | 1077 |
| Selective Attention | 840 | 1107 |
| Sustained Attention | 614 | 914 |
| Working Memory | 836 | 1144 |
